# MOFs as Potential Matrices in Cyclodextrin Glycosyltransferase Immobilization

**DOI:** 10.3390/molecules26030680

**Published:** 2021-01-28

**Authors:** Babatunde Ogunbadejo, Sulaiman Al-Zuhair

**Affiliations:** Department of Chemical and Petroleum Engineering, United Arab Emirates University, Al-Ain 15551, UAE; 201990035@uaeu.ac.ae

**Keywords:** cyclodextrins, cyclodextrin glycosyltransferase, immobilization, metal–organic frameworks

## Abstract

Cyclodextrins (CDs) and their derivatives have attracted significant attention in the pharmaceutical, food, and textile industries, which has led to an increased demand for their production. CD is typically produced by the action of cyclodextrin glycosyltransferase (CGTase) on starch. Owing to the relatively high cost of enzymes, the economic feasibility of the entire process strongly depends on the effective retention and recycling of CGTase in the reaction system, while maintaining its stability. CGTase enzymes immobilized on various supports such as porous glass beads or glyoxyl-agarose have been previously used to achieve this objective. Nevertheless, the attachment of biocatalysts on conventional supports is associated with numerous drawbacks, including enzyme leaching prominent in physical adsorption, reduced activity as a result of chemisorption, and increased mass transfer limitations. Recent reports on the successful utilization of metal–organic frameworks (MOFs) as supports for various enzymes suggest that CGTase could be immobilized for enhanced production of CDs. The three-dimensional microenvironment of MOFs could maintain the stability of CGTase while posing minimal diffusional limitations. Moreover, the presence of different functional groups on the surfaces of MOFs could provide multiple points for attachment of CGTase, thereby reducing enzyme loss through leaching. The present review focuses on the advantages MOFs can offer as support for CGTase immobilization as well as their potential for application in CD production.

## 1. Introduction

Cyclodextrins (CDs) are valuable compounds which have found applications in numerous fields, including the pharmaceutical, medical, food, and cosmetic industries. They are cyclic oligosaccharides consisting of six (α-CD), seven (β-CD), or eight (γ-CD) d-glucose units joined by glycosidic bonds to form a hollow truncated cone shape [1]. The importance of CDs stems from the amphibious nature of their structure, which exhibits a hydrophilic exterior that confers solubility in water and a hydrophobic interior cavity that forms inclusion complexes with various hydrophobic compounds [2].

CDs are produced by cyclization of dextrin or its derivatives, obtained during degradation of starch by cyclodextrin glycosyltransferase (CGTase). The product is a mixture of different major types of CDs (i.e., α, β, and γ) and negligible quantities of CDs with more than eight d-glucose units. However, the involvement of enzymes limits the production of CDs. One of the biggest challenges is the economic viability of the production process and the high cost of CGTase. To solve this issue, the biocatalyst must be effectively recovered and reused. Immobilization is the most common approach to achieve this, while maintaining the activity and enhancing the stability of the enzyme [3]. Notably, immobilization refers to physical confinement of an enzyme in a defined space [4]. In addition to increasing the enzyme recovery and reuse, immobilization of biocatalysts on suitable supports may also enhance their thermal and shear stability. Furthermore, immobilization enables efficient handling of the enzyme, adequate control of the reaction, and prevents contamination of the products.

The properties of the support play an important role in determining the success of the immobilization process; therefore, significant attention is paid to selecting an optimum support for the desired enzyme. Nevertheless, despite numerous advantages over soluble enzymes, the use of immobilized biocatalysts is associated with mass transfer limitations. Thus, the support should preferably be a porous material with a large surface area and void volume. The attachment of enzymes on such porous structures can be performed by physical and chemical adsorption. Physical adsorption is the most common immobilization technique owing to its simplicity and maintenance of the enzyme activity. However, enzymes adsorbed by such approaches are prone to leaching, which, with continuous use, leads to a decrease in the activity of the immobilized biocatalysts. Moreover, although chemical adsorption provides a stronger attachment to the support and, hence, results in resistance to leaching, the chemical bonds formed between the enzyme and support affect the activity of the biocatalyst. Other immobilization matrices such as sol-gel, hydrogels, and mesoporous silica have recently been suggested to overcome the leaching problem without affecting enzyme activity [5,6,7,8]. Nonetheless, the above materials exhibit low immobilization efficiency and high mass transfer. In addition, they cannot be used for bulky substrates due to the restricted access to the pores [5,6]. It is also noteworthy that immobilization in sol-gel takes place during sol-gel synthesis and subjecting the enzyme to harsh curing conditions results in reduced activity. These issues have been overcome by utilizing hydrogels instead of sol-gel; however, enzymes immobilized in hydrogels are prone to leaching upon swelling of the matrix [7]. Mesoporous silica displays a large surface area, theoretically making it an ideal immobilization material. Nonetheless, the presence of surface charges often leads to enzyme deactivation. Moreover, mesoporous silica also suffers from enzyme leaching [8]. In recent years, metal–organic frameworks (MOFs) have found use as an immobilization support for several enzymes and can as well be proposed as attractive alternatives to the aforementioned supports for CGTase. Compared to other immobilizing matrices, MOFs have been considered as promising materials due to the possibility of easy pore size modification, mild synthesis conditions, and desirable physico-chemical properties [9].

The present review discusses the use of immobilized CGTase for enhanced cyclodextrin production and highlights the potential of MOFs as new immobilization supports. Despite the clear evidence of the favorable characteristics of MOFs, to the best of our knowledge, there is no report in the literature showing MOF usage for CGTase immobilization. The critical discussion presented in the review paves the way for researchers to investigate the effectiveness of using MOFs in this very important application.

## 2. Cyclodextrin Glycosyltransferase

Enzymes are typically classified into six groups based on their function, namely hydrolases, lyases, isomerases, ligases, transferases, and oxidoreductases. Transferases catalyze the transfer of functional groups between molecules. CGTases (EC 2.4.1.19) belong to this category and have the ability to catalyze four different types of reactions, namely cyclization, coupling, hydrolysis, and disproportionation [10]. 

CGTases are extracellular enzymes obtained only from bacterial cells. They exhibit certain functional similarities to amylases, which hydrolyze starch or starch derivatives into linear products. Hence, CGTases that are thermally stable can be employed for solubilization of starch [11]. These biocatalysts are classified into α-, β-, and γ-CGTases based on the major CD produced in the initial phase of the reaction between the enzyme and starch [12]. 

### Sources and Properties

Table 1 shows examples of different enzyme sources and their optimum growth conditions. The bacteria used for obtaining CGTase for the production of CD are selected based on the preferred type of CD. For instance, CGTase produced from *Bacillus pseudalcaliphilus* 8SB has been reported to exhibit no α activity, high β activity, and low γ activity [13].

The molecular weight of CGTase can be taken to be 77.24 kDa based on the work of Joost et al. [25], which gives an approximate size of 5.62 nm based on the equation developed by Harold [26]. The sizes of CGTase and substrate must be taken into consideration when deciding which matrix or immobilization method to use. Identifying the inefficiencies present in the matrices used so far can help in making adequate decisions on the types of MOFs that can improve CGTase immobilization.

## 3. CGTase Immobilization

**Figure 1:**
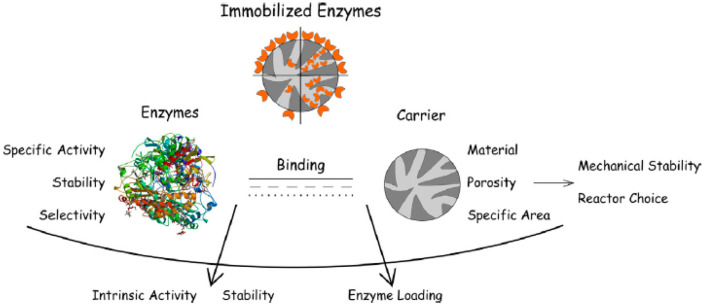
Summary of enzyme immobilization and the desired enzyme and support properties [28].

The necessity for appropriate enzyme handling, storage, and reuse is driving research concerning immobilization of biocatalysts on different supports. For the economic viability of any biochemical process, the cost of enzymes should not be more than a few percent of the total cost of the production; thus, the possibility of biocatalyst reuse is important [7]. Traditionally, enzymes are lyophilized, i.e., freeze-dried; however, this may lead to significant distortion of the enzyme structure [27]. Immobilization of enzymes on supports enables better access for the substrates as the biocatalysts are dispersed, thus increasing the available surface area. The support should preferably be inert to the enzyme and possess microbial resistance. Additionally, it should not pose diffusional problems to the enzyme’s substrate. The desired properties for the enzyme and support are summarized in Figure 1 [28].

To efficiently utilize CGTase for the production of CD, different supports have been used for its immobilization in the literature. Table 2 summarizes the previously reported supports for CGTase immobilization. 

### 3.1. Supports Used for CGTase Immobilization

Nanomaterials have been employed as supports for CGTase. For example, cellulose nanofibers (CNFs) made from kenaf bast fiber were used for immobilization of CGTase using chemical coupling with 1,12-diaminododecane as a coupling agent [29]. The immobilized enzyme was added to a 50 g/L soluble starch solution and incubated at an optimum temperature of 70 °C. HPLC analysis of the product revealed a gradual increase in the yield of α-CD, which reached a maximum of 69%. The performance of the immobilized CGTase prepared at higher microwave power levels led to CNFs with a smaller diameter (higher surface area), resulting in better interaction between the coupling agent and the –OH group present on the cellulose, observed at 3400–3200 cm^−1^. The covalent attachment of CGTase to the ligand ensures that the enzymes gain rigidity by reducing the chances of conformational changes, thus resulting in better stability. This could be observed in the thermal stability, which shifted from 60 °C for free CGTase to 70 °C for immobilized CGTase, and the retained activity after 10 cycles was 68% [29]. The maximum binding efficiency, after several modifications on both the process and the CNF synthesis parameters, was 72%.

In another study, Fe_3_O_4_ nanoparticles functionalized with polydopamine (PDA) were used [13]. It has been reported that PDA contains various surface functional groups such as amino and catechol which influence enzyme immobilization [30,31]. The immobilized biocatalyst was mixed with 3 mL of a 1% potato starch solution. The reaction was conducted at 55 °C and the maximum yield of β-CD was 88.9%. It is noteworthy that the immobilized enzyme retained 19% of its initial activity after nine cycles, which showed that the attachment of CGTase to the functional groups present on PDA might not be enough for use in industrial set-up.

Commercially available Eupergit C and Eupergit C 250 L, which are epoxy-activated acrylic beads a with difference in their pore sizes and oxirane groups, were used for CGTase immobilization [32]. The average pore size in Eupergit C is 10 nm, making it mesoporous based on the classification of the International Union of Pure and Applied Chemistry (IUPAC), and it has an oxirane density of 600 µmol/g. Eupergit C 250 L, on the other hand, is macroporous (average pore size 100 nm), but with a lower oxirane density of 300 µmol/g [33]. The immobilization mechanism of enzymes on Eupergit beads has been proposed to follow two steps [34]: Firstly, there is physisorption on the support by hydrophobic interaction, which brings the amino and thiol groups present on the enzyme’s surface close to the oxirane group. Then, these groups react with the oxirane group via nucleophilic attack to form very stable C-S and C-N bonds. Therefore, it is expected that Eupergit beads should offer minimum enzyme loss when used as immobilization supports. The percentage of bound protein for Eupergit C 250 L was 72% compared to 81% observed on Eupergit C [32]. As Eupergit C contains more oxirane groups, this leads to better retainment of CGTase and can also promote multipoint attachment to produce a more stable enzyme/support matrix. The reusability studies showed that 40% of the initial CGTase activity was retained after 10 cycles of 24 h each. Despite these advantages of Eupergit C over Eupergit C 250 L, its mesopores will pose diffusional limitations, especially for a bulky substrate, e.g., starch, in the production of CD using CGTase.

In addition, Schoffer et al. described immobilization of β-CGTase on glutaraldehyde pre-activated silica, functionalized with 3-aminopropyltrimethoxysilane (APT) [35]. Although the immobilization yield was high (above 96%) as a result of functionalization, the efficiencies were very low, between 3% and 5%. A possible cause of this could be the mesoporous nature of the silica used preventing the substrate from accessing the active sites. At an optimal temperature, pH, and reaction time, the immobilized enzyme resulted in the production of 4.9 mgmL^−1^ of α-CD, 3.6 mgmL^−1^ of β-CD, and 3.5 mgmL^−1^ of γ-CD. Moreover, porous glass beads (e.g., Trisoperl) functionalized with APT have also been employed in the presence of glutaraldehyde as the cross-linker. The catalytic activity of the immobilized enzyme was studied in a reaction involving a 2.5% (*w*/*v*) starch solution. A maximum CD yield of 85% was obtained at a temperature of 37 °C and pH of 6.0 [36]. A lag phase of 10 min was observed before the reaction started using the immobilized CGTase, supporting the earlier assertion that diffusional barriers exist.

Furthermore, CGTase was previously also immobilized by covalent attachment on polyvinylchloride aminated with three different dialkylamines using glutaraldehyde. The enzymatic activity reached 121 U/g in a reaction involving a 5% (*w*/*v*) starch solution. Stability studies showed that immobilized CGTase retained 85% of activity after 14 cycles of batch operation. It was demonstrated that the amount of retained activity depended on the length of the spacer, i.e., the dialkylamine group and glutaraldehyde [37]. 

On the other hand, on a macroreticular hydrophilic resin (e.g., FE 4611) containing a primary amine, the optimum pH of the immobilized enzyme was shown to range between pH 6.0 and 8.0, with a maximum β-CD yield of 14% [38]. CGTase was also successfully immobilized on calcium alginate beads [39]. Under optimized conditions, 43% immobilization efficiency was achieved using a starch concentration of 3.5% (*w*/*v*). The immobilized enzyme exhibited good activity of 2760.4 U/mL. Notably, the CGTase immobilization yield reached nearly 100% after 5 h at 25 °C when glyoxyl-agarose was utilized as the support at pH 10. Using immobilized CGTase on a 1% soluble starch solution at 85 °C, a β-CD yield of 85.4% was obtained at a two-fold higher rate than that observed for the free enzyme [40].

Since CGTase from different bacterial sources displays various optimum temperatures as shown in Table 1, a number of ionic interactions and disulfide bonds present in the structures of different sources of CGTase were examined [41]. All of the studies exhibited a similar number of disulfide bonds; however, thermally stable enzymes displayed more ionic interactions. Hence, disulfide bonds are not responsible for the thermal stability of CGTase [42]. The attachment of the CGTase to the support, depending on the functional group on the support, could be responsible for this observed phenomenon.

From the supports used in the literature for CGTase immobilization, it is evident that apart from differences in physical characteristics such as the pore diameter, particle size, and mechanical strength, the performance of the CGTase/support depends on the type and density of the functional group used, the length of the coupling agent (for covalent bonding), and the pore network of the support. The surface area of the support, which depends on the pore diameter and particle size, significantly affects the capacity for CGTase binding. It is noteworthy that porous supports such as agarose or Trisoperl displayed better immobilization yield (thus, increased CD yield), particularly in the presence of hydrophilic moieties. For example, utilizing agarose, a highly porous matrix with hydrophilic properties, resulted in an 85.4% yield of β-CD, which was comparable to that achieved using Trisoperl and Fe_3_O_4_@PEI-PDA (Table 2). For more optimal utilization of porous supports, better control of the pore size distribution, such as by utilizing supports with hierarchical pore networks, will likely give better results. This would improve the diffusional limitation and enhance the production of CD. 

**Table 2:** Properties of CGTase immobilized on different supports for cyclodextrin (CD) production.

Support	Source of CGTase	Optimum pH	Optimum Temperature (°C)	Activity of Immobilized Enzyme (U/g-Support)	Maximum Yield of CD (%)	Reusability Studies (% of Initial Activity Retained after Cycles)	Ref.
Physical Adsorption							
Polyvinylidene difluoride hollow fiber	*Bacillus lincheniformis*	7.0	25	n.r	69.37	n.r	[43]
Covalent Attachment							
Cellulose nanofiber	*Bacillus macerans*	n.r.	70	159.34	69 (α)	68% after 10 cycles	[44]
Trisoperl (activated porous glass)		5.1	48	3.0	~85 (β)	68% after 20 cycles	[36]
Aminated polyvinylchloride (PVC)		6	75	121	15.6	85% after 14 cycles	[37]
Fe3O4@PEI-PDA	*Bacillus pseudalcaliphilus*	6.0	55	300	88.9 (β)	19% after 9 cycles	[13]
Resin (FE 4611)		6–8	~58	≤ 2	14	n.r.	[38]
Glutaraldehyde-pre-activated silica	*Thermoanaerobacter* sp.	4.0–8.0	n.r.	101.73	n.r.	n.r.	[35]
Glyoxyl-agarose		6.0	85	27.38	85.4 (β)		[40]
Functionalized magnetic double mesoporous core-shell silica	*Amphibacillus* sp.	8.5	55	n.r.	n.r.	56% after 10 cycles	[45]
Entrapment							
Calcium alginate beads	*Bacillus maceran*	7.5	60	n.r.	n.r.	75% after 7 cycles	[46]
	*Aspergillus oryzae*	4.0	40	2760.4 U/mL	n.r.	57% after 12 cycles	[39]

### 3.2. Immobilization Technique

The most commonly used enzyme immobilization approaches include surface adsorption, covalent binding, encapsulation, and cross-linking. Selection of the method depends on the properties of the enzyme and support as well as on the potential application of the immobilized biocatalyst.

Surface adsorption occurs through a physical interaction between an enzyme and support. It is achieved by soaking the support in a buffered solution of the enzyme for a suitable incubation time. Alternatively, the biocatalyst solution can be allowed to dry on the support surface before washing away unattached enzymes [47,48]. The reversible nature of physical adsorption enables the removal of immobilized enzymes from supports under mild conditions upon deterioration of the enzymatic activity [49]. Nevertheless, the weak forces holding the enzymes make them susceptible to leaching from the support when subjected to industrial conditions.

Ionic and covalent binding of the enzyme to the support is stronger than the physical adsorption described above. It offers enhanced enzyme stability; however, the presence of chemical bonds may affect the activity of the attached biocatalyst, which is a major disadvantage of this approach [7]. Generally, compared with a free enzyme, a reduction in activity is observed when an enzyme is immobilized on a support due to several factors, including protein crowding, biocatalyst inactivation, stearic hindrance, and enzyme orientation.

Enzyme encapsulation is a method of immobilization whereby the enzyme is confined within a porous support. Encapsulation can be achieved either by impregnating the enzyme onto the synthesized support or during support synthesis, referred to as biomimetic encapsulation [50]. A mechanochemical method of encapsulating enzymes have also been demonstrated [51]. The entrapped enzyme is not actually physically attached to the support; however, its ability to diffuse out is restricted [52]. Enzyme entrapment is fast and involves mild conditions. Moreover, the enzyme is not chemically interacting with the support and the possibility of denaturing is lower. Encapsulating an enzyme could enhance its catalytic performance as enzyme structures tend to change upon encapsulation as depicted in the work using cytochrome c (Cyt-c) [50,53,54]. Nonetheless, this approach suffers from mass transfer limitations, as the access of the substrate to all active sites might be restricted [55].

More recent immobilization techniques include cross-linked enzyme aggregates (CLEAs) and cross-linked enzyme crystals (CLECs). These methods are typically called carrier-free immobilization approaches as there is no requirement for any supports [7]. As described above, immobilization of enzymes on supports involves attachment of biocatalysts on the surface of suitable materials. On the other hand, in the case of cross-linking, the enzyme exhibits greater stability because it is stabilized by links in a 3D structure [56]. Consequently, cross-linked enzymes usually display enhanced mechanical stability, ability to withstand shear stress, and improved high-temperature tolerance compared to other immobilized enzymes.

The formation of CLEAs involves the generation of enzyme aggregates in the presence of salts, non-ionic polymers, or organic solvents, followed by cross-linking using bifunctional (e.g., glutaraldehyde) or poly-functional (e.g., aldehyde–pectin, aldehyde–dextran, or aldehyde–starch) chemical agents without the need for a support [57]. This approach offers various advantages over other immobilization methods, including simplicity as well as thermal and operational stability of the aggregates. Importantly, it can easily be applied to more than one enzyme at a time. The effects of cross-linking agents on the activity of CGTase CLEAs have been previously investigated [58]. Nevertheless, the cross-linking technique is also associated with several limitations. Even at an optimum concentration of the cross-linking agent, i.e., glutaraldehyde, the activity of the recovered enzyme was determined at <10%, while the aggregate activity loss was established at >80%. The observed low activity recovery was attributed to diffusional resistance of the bulky starch substrate or inadequate enzyme cross-linking, leading to increased loss of the enzyme, thus resulting in low activity recovery.

On the other hand, CLECs are solid crystalline particles, which are insoluble in organic solvents and water. They are prepared by precipitating enzymes into microcrystals, which is followed by a cross-linking step. The lattice interactions in the microcrystals provide additional stability for the biocatalysts. The advantages and disadvantages of various immobilization techniques are summarized in Table 3. 

From the various methods used for CGTase immobilization, covalent attachment has shown to offer the best immobilization yield with relatively higher activity retainment.

## 4. Metal-Organic Frameworks

In recent years, MOFs have been utilized in various fields; therefore, their application as supports for enzyme immobilization has attracted significant attention. MOFs are formed by linking metal ions and organic linkers into well-defined three-dimensional porous solids [66]. The surface area of these materials ranges from 1000 to 10,000 m^2^/g, surpassing that of other known porous structures [67]. The stability of MOFs depends on the strength of the metal–organic linker coordination bond [68,69]. Notably, nearly all metal atoms in their stable oxidation states can be used for the synthesis of MOFs. The coordination number of employed metals defines the possible molecular geometry, e.g., linear, planar, pyramidal, or octahedral [70]. 

Commonly used organic linkers include carboxylates, sulfonates, imidazolate, amines, and their derivatives. The functional groups on the organic linkers in MOFs must be carefully selected as they provide the necessary interaction sites for the enzyme. Appropriate functionalities minimize leaching and improve stability [71]. To investigate this in more detail, the interactions between microperoxidase 11 (MP-11) and mesoporous Tb-MOF were studied using Raman spectroscopy. The presence of a π–π interaction between the organic component of the examined MOF and the heme unit of MP-11 was established. However, this interaction was missing when mesoporous silica was used instead of Tb-MOF [72]. Generally, a ligand is said to be flexible if it can rotate around a single bond. It is noteworthy that during the selection of organic linkers, rigid organic molecules are preferred over flexible ones. Rigid molecules aid the formation of crystalline MOFs exhibiting good thermal and mechanical stability and specific topology [73,74]. Moreover, both charged and neutral compounds can be used as ligands for MOF synthesis; however, positively charged molecules are used less frequently. This is predominantly due to the low affinity of positively charged moieties for the formation of bonds with metal cations, i.e., the required charge balance cannot be achieved [75]. The metal centers in the coordination spheres created by the metal ions in MOF structures are usually protected from the reactants by bulkier organic linkers [76]. 

**Figure 2:**
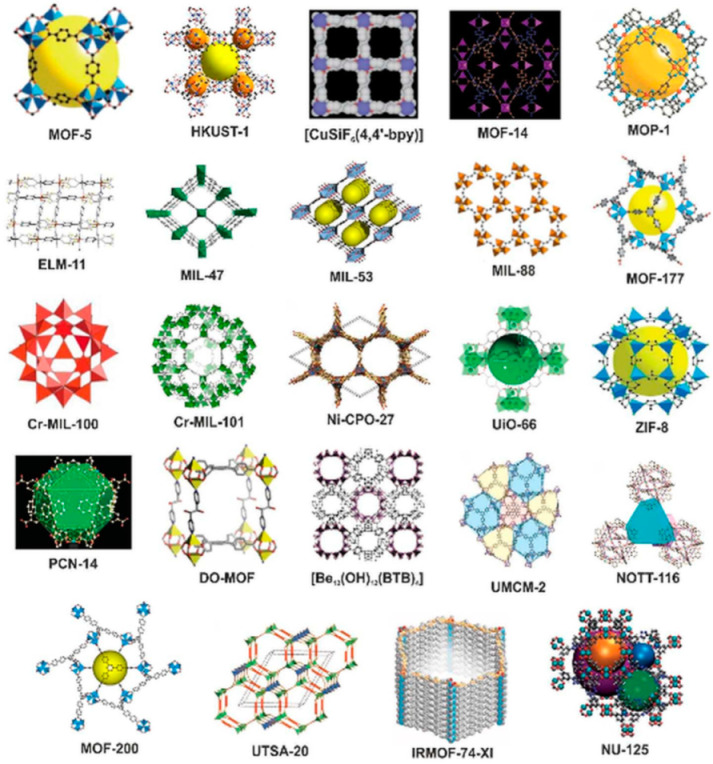
Examples of typical metal-organic framework (MOF) structures. Reproduced from Ref. [77] with permission from the Royal Society of Chemistry.

The availability of different metal ions and organic linkers results in the formation of MOFs with different physicochemical properties. Examples of typical MOF structures are demonstrated in Figure 2.

A more recent subclass of MOFs is biological MOFs (bio-MOFs), which introduce biomolecules in the formation of the porous material. Apart from the general properties offered by MOFs, bio-MOFs offer much-needed properties, especially in biological application, where toxicity [78], efficacy, and stability must be critically controlled [79]. Most bio-MOFs are constructed using a biomolecule as the ligand, such as proteins, amino acids, peptides, and cyclodextrins, or any other biorelevant organic linker which can bind to bioactive metal nodes [79,80,81]. Examples of successfully synthesized bio-MOFs include Co-Cys, made from cobalt and cystine [82], and silver-based phosphaadamantane (Ag-PTA) [83]. Silver is very useful in bio-MOF synthesis due to its recognized antimicrobial action.

### Types and Properties

Various types of MOFs have been synthesized for application in enzyme immobilization. The majority of the investigated MOFs can be synthesized under ambient conditions. These include zeolite imidazolate frameworks (ZIFs), terbium benzene-1,4-dicarboxylate (Tb-BDC), Materials of Institute Lavoisier (MILs), and Hong Kong University of Science and Technology MOFs (HKUST). Synthesis under ambient conditions enables in-situ encapsulation of the enzyme, also known as co-precipitation. During the process, the biocatalysts are not subjected to harsh conditions; therefore, their activity is not affected.

Synthesis of MOFs is often conducted in liquid phase by mixing different solutions containing the chosen metal and organic linkers, e.g., at room temperature. The available synthesis methods include solvothermal [84], microwave-assisted [85], sonochemical [86], mechanochemical [87], electrochemical [88,89], and direct evaporation (also known as slow diffusion) approaches [90,91,92]. A summary of different MOF preparation methods as well as their advantages and disadvantages is demonstrated in Table 4.

Solvothermal approaches are the most commonly employed methods for the production of MOFs. In solvothermal synthesis, the reactants are subjected to temperatures in the range of 100–240 °C and pressures up to 105 kPa. In addition, polar solvents with high boiling points are typically utilized, and the reaction time ranges from 6 h to several days. Sealed vessels or Teflon-lined autoclaves are used for the reaction [104]. Hydrothermal synthesis can be taken as a special class of solvothermal technique, involving the use of aqueous solvents, usually water, at elevated temperature and pressure [105]. Its advantages in MOF synthesis include growth of microcrystalline phases during MOF synthesis, utilization of water, considered as a green solvent, and high reactivity of the reacting species [106,107]. The hydrothermal route can also be used to obtain MOFs with extended channel systems [108]. Due to the elevated synthesis conditions, encapsulation of CGTase within the pores of MOFs in a one-pot synthesis might not be feasible, as the harsh conditions might cause structural damage to the enzyme. A post-synthesis modification of the MOFs remains the best immobilization route for these synthesis approaches.

To accelerate the process, microwaves or ultrasound waves are utilized in microwave-assisted and sonochemical methods, respectively. In microwave synthesis, the reaction medium heats up due to the effect of the applied oscillating electric field on the permanent dipole moment of the molecules present in the medium, resulting in molecular rotations and rapid heating [109]. The usefulness of microwave synthesis largely depends on the dipole moment of the solvent molecule. Solvents with large dipole moments, such as dimethylformamide, DMF (dipole moment = 3.86 D), are preferred [110]. On the other hand, in sonochemical synthesis, the increased heating rate is a consequence of acoustic cavitation, which is the formation and collapse of bubbles by ultrasound waves, typically between 20 kHz and 1 MHz. This phenomenon results in an increased heating rate (>1010 K/s), temperature (as high as 5000 K in gas-phase reaction zones), and pressure (up to 1000 bar) [111,112,113]. Nonetheless, both of the above accelerated methods lead to the formation of MOFs exhibiting small crystal sizes, which range between 10 nm and 50 µm. Unlike other approaches, mechano-chemical synthesis is characterized by the absence of solvents. In the process, the intramolecular bonds between the metal salts and the organic linker molecules are subjected to mechanical breakage using a ball mill, which results in a chemical transformation and the formation of the desired MOFs [91,114]. Furthermore, electrochemical synthesis is similar to the solvothermal method. However, instead of using metal salts, metal ions are supplied from the dissolution of the anode. The metal ions then react with the dissolved linker molecule present in the reaction medium [114]. Direct evaporation, or slow diffusion, is also comparable to the solvothermal approach. In this case, however, no external energy is needed. The metal salts and organic linkers are mixed and the solvent gradually evaporates from the reaction solution at room temperature [115]. When selecting a suitable method, considering the reaction time and the amount of solvent needed for the synthesis of MOFs is necessary. In all approaches, the structural units self-assemble into ordered metal-organic coordination bonds to form the structural frameworks of MOFs. The simplest method reported to date is direct evaporation, which does not necessitate the use of any external energy sources. However, the approach requires long synthesis times, which range from a few hours to several days.

**Figure 3:**
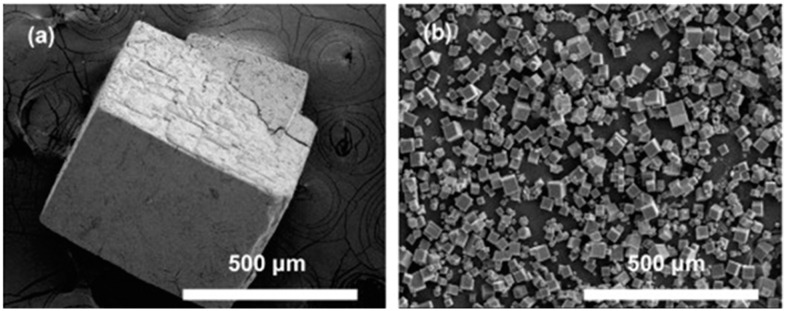
Scanning electron microscopy (SEM) images of MOF-5 crystals obtained using (**a**) conventional and (**b**) microwave-assisted approaches [118].

The rate of reaction can be significantly increased using microwave synthesis, which results in rapid achievement of high temperatures in localized zones. Examples of MOFs that have been synthesized employing this method include IRMOF-1 [96], HKUST-1 [116], and MIL-100-Cr [95]. To understand the mechanism of microwave synthesis, the rate enhancement was studied during the preparation of HKUST-1 [116]. It was found that the reaction rate enhancement was the result of an increase in the nucleation rate and not the crystal growth rate. In contrast, the outcomes of the investigation involving MIL-53 (Fe) demonstrated that both nucleation and growth rates contributed to the observed enhancement in the rate of reaction [117]. Despite the increased rate of reaction, using microwave-assisted heating results in significantly smaller crystals compared to other methods. For instance, the synthesis of MOF-5 utilizing the direct evaporation and microwave-assisted methods resulted in similar cubic-shaped MOFs; however, the crystal size of the microwave-heated material was approximately 20 times smaller (Figure 3) [118]. 

Consequently, based on the promising results obtained by immobilizing CGTase on functionalized supports as highlighted in Section 3.1, improvement in CGTase immobilization is expected using MOFs based on the numerous advantages that have been highlighted. The presence of both organic (i.e., the linker) and inorganic (i.e., metal nodes) components in MOFs could promote several interactions between the support and various functional groups in CGTase. The organic linker can be modified to generate interaction sites for CGTase, thereby minimizing leaching and enhancing stability. In addition, the ordered, crystalline, and multi-dimensional structure of MOFs could ensure a protective environment for CGTase and prevent activity loss due to denaturing factors. This would further reduce leaching of the biocatalyst and limit mass transfer problems. Nevertheless, careful selection of appropriate MOFs is essential to ensure the presence of suitable cavities capable of accommodating CGTase [119]. In this regard, MOFs exhibiting hierarchical structures are promising. The large channels in these materials could be used for immobilization of CGTase, while the smaller channels would remain available for substrate and product diffusion, minimizing diffusional problems [119]. Mesopores can be created in MOFs structure through different methods, mostly by templating, a recently observed method is via induced structure defect using microfluidic flow [120]. The mixing pattern during synthesis was modified which led to loss of metal atoms, resulting in change in the MOF porosity, thereby resulting in better enzyme activity.

Different MOFs with hierarchical pore networks have already been synthesized by various researchers that could be adapted for limiting the effect of diffusion in CGTase immobilization. Mondloch et al. [121] described the synthesis of hierarchical zirconium-based MOFs (NU-1000) which contain windows connecting 3.1-nm hexagonal channels with triangular channels having an edge length of 1.5 nm. In making hierarchical MOFs, the nucleation and growth processes must be well controlled [122,123]. Also, MOFs architecture can be controlled in-situ. This has been demonstrated with ZIF-8, transforming its three-dimensional microporous structure into two-dimensional mesoporous layer using a modulator [124].

The large surface areas of MOFs could result in higher CGTase loading capacity, providing more active sites for substrate transformation into CD. Lastly, the ability to tune the properties of an MOF, most importantly the hydrophilic or hydrophobic nature of its surface, would ensure that the free cysteine residue in the enzyme could be used for binding to the support, leading to better biocatalyst stability.

## 5. Conclusions

In this review, CGTase immobilization on various functionalized supports was discussed. Covalent attachment was identified as the best immobilization technique for CGTase, with density of the functional group, length of the coupling agent, and type of pores present in the support affecting the reusability of the matrix and diffusion of both reactant and product when used in CD production. MOFs have been shown as robust supports for enzyme immobilization, exhibiting enhanced biocatalytic properties compared to conventional supports. The increasing number of new MOF structures will undoubtedly lead to utilization of these materials in various fields. Although MOFs have been employed for the immobilization of several different enzymes, their superior properties have not been applied for CGTase immobilization to produce CD. To achieve this, the interactions between CGTase and MOF components must be investigated and understood. Better knowledge of such interactions could lead to enhancement in enzyme loading, stability, and reusability, particularly in industrial settings. As immobilized enzymes display different activity trends from those of free biocatalysts, further research is needed to determine the effects of CGTase immobilization on MOFs on the activity of the enzyme. Moreover, the affinity of MOFs for CGTase requires evaluation. Among the possible conventional immobilization techniques, covalent attachment of enzymes has been shown to result in more stable and reusable biocatalysts. Nevertheless, the high cost and difficult regeneration of the support currently limit the application of this technique. Thus, improvements are needed to utilize this approach for industrial production of CD.

## Figures and Tables

**Table 1:** Cyclodextrin glycosyltransferase (CGTase) sources and optimum growth conditions.

Bacteria	Type of CGTase	Optimum Condition	Reference
*Bacillus licheniformis*	α-CGTase	40 °C, pH 6.0–8.0	[14]
*Bacillus circulans*	β-CGTase	56 °C, pH 6.4	[1]
*Bacillus* sp.	β-CGTase	55 °C, pH 5.0	[15]
*Bacillus agaradhaerens*	β-CGTase	55 °C, pH 9.0	[16]
*Bacillus megaterium*	β-CGTase	60 °C, pH 7.2	[17]
*Bacillus subtilis*	γ-CGTase	65 °C, pH 8.0	[18]
*Bacillus firmus* strain 290-3	β/γ-CGTase	60 °C, pH 6–8	[19]
*Paenibacillus macerans*	α-CGTase	45 °C, pH 6.0–10	[20]
*Thermoanaerobacterium thermosulfurigenes*	α-CGTase	80–85 °C, pH 4.5–7.0	[21]
*Geobacillus thermoglucosidans*	β-CGTase	65–70 °C, pH 5.5	[10]
*Brevibacillus brevis* strain CD162	β/γ-CGTase	55 °C, pH 8.0	[22]
*B. macorous strain* WSH02–06	γ-CGTase	50 °C, pH 6.5	[23]
*Brevibacterium* sp. strain 9605	γ-CGTase	45 °C, pH 10	[24]

**Table 3:** Summary of conventional immobilization techniques.

Immobilization Method	Binding Characteristics	Advantages	Disadvantages
Physical adsorption [59,60]	Weak bonds by either van der Waals or ionic interactions	Simple/cheap Little or no conformational change in the enzyme Ease of regeneration Wider selection of support	High enzyme desorption/leaching
Covalent binding [61]	Chemical attachment between functional groups on support and enzyme	Low enzyme leaching Enhanced enzyme stabilization	Difficulty in regenerating enzyme/support Reduced enzyme activity
Entrapment/encapsulation [62,63]	Inclusion of enzyme within the supports structure	High enzyme loading Low enzyme leaching Little or no conformational change in the enzyme	High mass transfer limitation Inactivation of enzyme during encapsulation
Cross-linking [64,65]	Aggregate/cluster of enzyme cross-linked by a functional reactant	Enzyme stabilization without support	High mass transfer limitations Loss of enzyme activity Less useful in packed bed reactors

**Table 4:** Summary of MOF synthesis methods.

Synthesis Method	Advantages	Disadvantages	Examples of MOFs
Solvothermal	Ease of technology transfer to the industry Crystal growth can be controlled Wide temperature range	High operating cost Long synthesis time	ZIF-95 [93] ZIF-78 [94]
Microwave-assisted	Reduced crystallization time Energy efficient Ease of controlling reaction conditions Particle size can be controlled from precursor concentration	Difficult to implement in the industry Isolation of single crystals is nearly impossible	VSB-1, VSB-5 [95] IRMOF-1, IRMOF-2, IRMOF-3 [96] Zr-UiO-66 [97] Hf-UiO-66
Sonochemical/Ultrasonic	Can achieve homogenous crystal size and morphology Can be used to isolate pure phase	Breakage of large single crystals needed for diffraction studies	TMU-46, TMU-47, TMU-48 [98]
Mechanochemical	Only mechanical forces are needed Extreme operating conditions are avoided Solvent-free	Difficulty in obtaining a single crystals for diffraction studies Secondary phases usually present in product	Copper isonicotinate Cu(INA)_2_ [99] Copper benzenetricarboxylate Cu_3_(BTC)_2_ [100] Cd(II)-based MOFs [87]
Electrochemical	Ease of industrial application Short synthesis time Uses current and voltage to control morphology	ew MOFs have been synthesized to date	UiO-66 [101] Cu_3_(BTC)_2_ [102]
Slow diffusion	Preparation of large single crystals Ambient or low temperature required	Synthesis could take several days Minute quantity of product	Zn_3_(BDC)_3_.6CH_3_OH [103]
